# For a hepatitis-free future

**DOI:** 10.1016/j.lanwpc.2021.100234

**Published:** 2021-07-26

The theme of World Hepatitis Day on July 28 this year is “Hepatitis can't wait”, conveying the urgency of efforts needed to eliminate hepatitis as a threat to public health by 2030. Hepatitis B and C are the most discussed among the five subtypes of viral hepatitis because they have a higher prevalence and more severe health consequences than the other subtypes. Nowadays, hepatitis B virus infection can be effectively prevented by vaccination and fully controlled with a daily pill; hepatitis C virus infection is the first and only chronic disease to be curable by direct-acting antiviral regimens. With these great achievements in the battle against viral hepatitis it seems we are approaching the goal of hepatitis elimination, but that is not the reality in the Western Pacific region.

In 2016, an estimated 325 million people worldwide had hepatitis B or C virus, among whom, the Western Pacific region accounts for 40% of the total number and has the highest hepatitis B prevalence. Moreover, six countries in the region are among the top ten countries in the world with the highest incidence of liver cancer, a long-term sequela of chronic hepatitis B and C virus infection. Furthermore, the Western Pacific region has the highest number of viral hepatitis related deaths per year, accounting for approximately 39% of the global mortality due to viral hepatitis.

In 2016, WHO launched the Global Hepatitis Programme with the aim to eliminate viral hepatitis as a public health threat by 2030, to reduce new hepatitis infections by 90%, and to reduce deaths by 65%. The Western Pacific region has made good progress in hepatitis B immunisation (84% hepatitis B vaccine coverage *vs* 43% globally). However, as of 2016, only 17% of people with hepatitis B virus and 21% of people with hepatitis C virus in the region were diagnosed, and only 20% and 9% of those diagnosed, respectively, received treatment, making low coverage of testing and treatment the major obstacles to be addressed to achieve the 2030 elimination goal. As mentioned in the white paper launched by the World Hepatitis Alliance in 2018, the main barrier to hepatitis testing is poor hepatitis knowledge among the public and health-care professionals. Similarly, knowledge about the prevention and treatment of viral hepatitis and liver diseases is low among stakeholders in many parts of the Western Pacific region, especially in the lower-income countries and areas in the region (eg, Laos, Mongolia, and Vietnam) where inequalities exist in medical resources and financial support. As for access to treatment across the Western Pacific region, the high price of medicine remains the major challenge.

To overcome these challenges and to reach the goal of hepatitis elimination by 2030, the Western Pacific region needs to further raise public awareness and knowledge, to scale-up easily accessible testing, and to provide affordable medicines and timely treatments. To this end, countries and areas in the region should follow the WHO hepatitis guidelines covering a wide range of contexts, such as prevention, testing, care and treatment, and the guidance for country validation of viral hepatitis B and C elimination released in June, 2021. From another perspective, as the Western Pacific region now has more than 35 members across ten countries and areas that have joined the World Hepatitis Alliance—a global patient-led and patient-driven hepatitis alliance—the involvement of civil society and the affected community has supplied new power to hepatitis elimination by bringing forward fundamentally important recommendations and experiences from the perspectives of patients to policy makers. Altogether, these inputs could greatly enhance the effectiveness of strategies for hepatitis elimination.

In a recent Article from The Lancet Gastroenterology & Hepatology, Iceland has become the first country to claim success in reaching the 2030 hepatitis elimination goals, having more than 90.0% of the infected population diagnosed, 95.3% of diagnosed infections being treated, and 90.2% being cured, making the WHO goal of hepatitis elimination a reality. The journey to hepatitis elimination in the Western Pacific region is still long; however, hepatitis cannot wait and should not wait.

*The Lancet Regional Health – Western Pacific*

